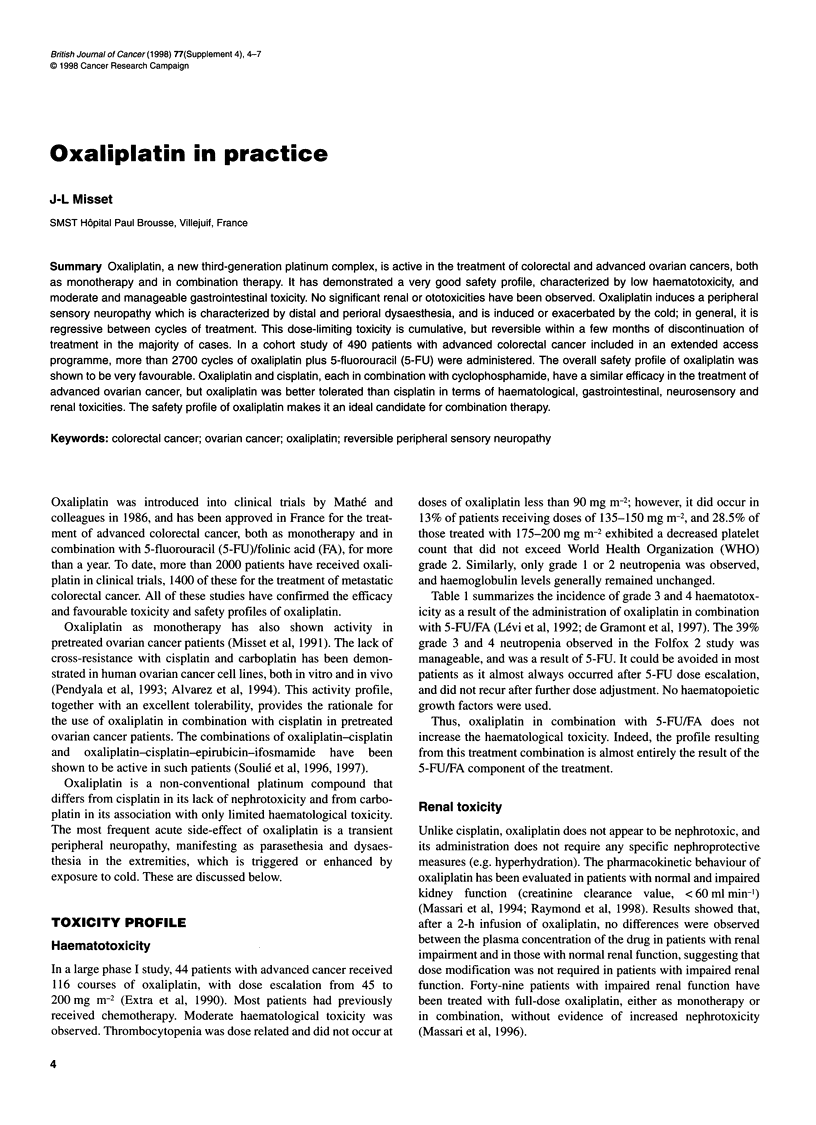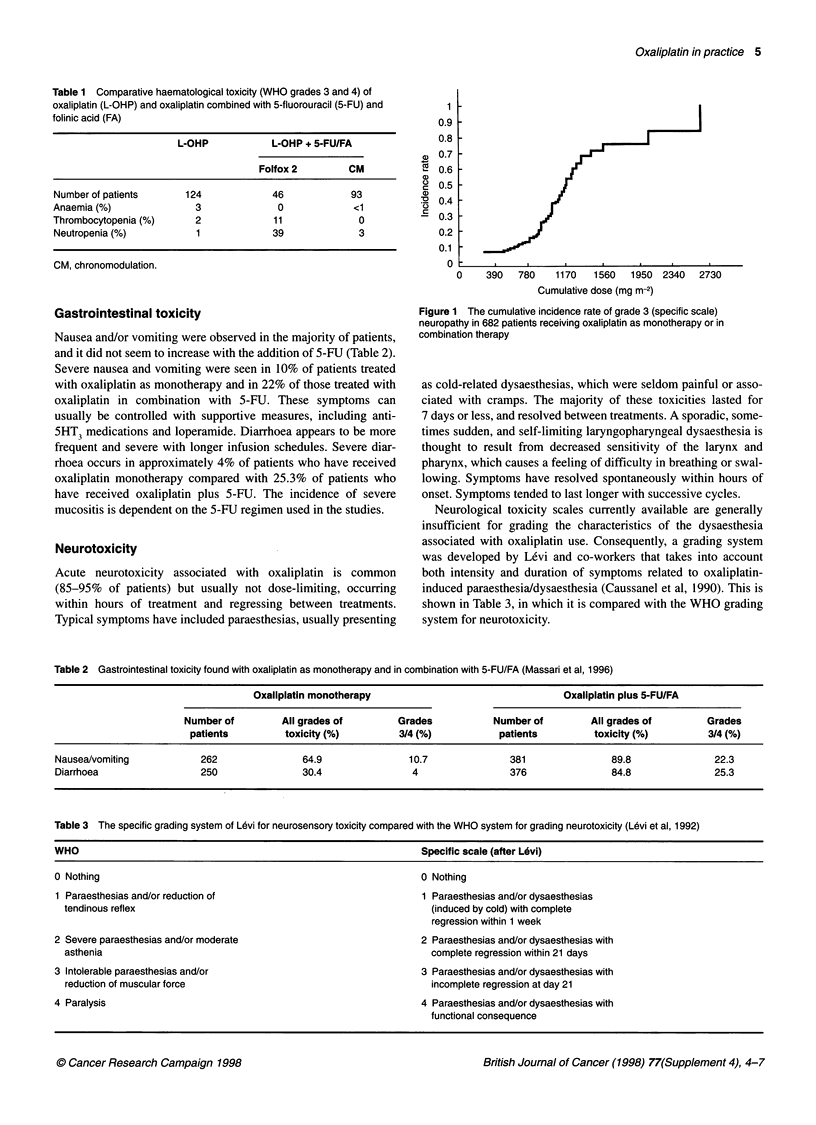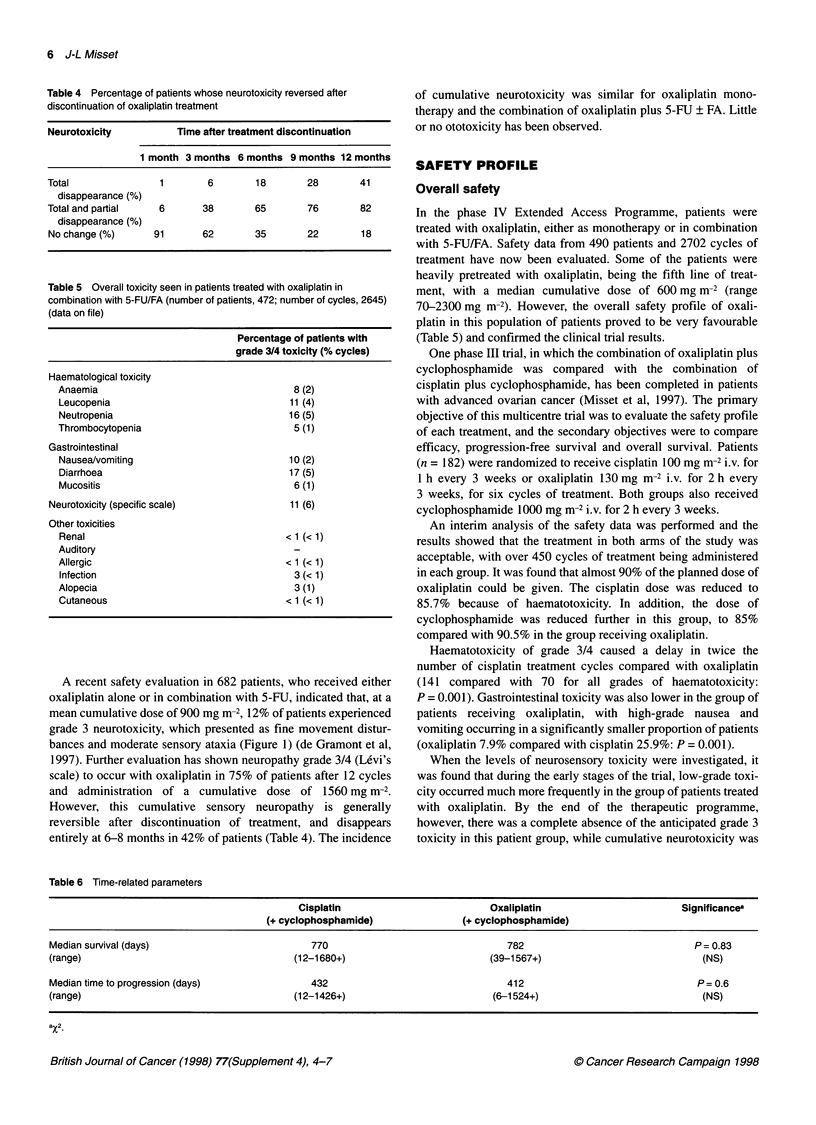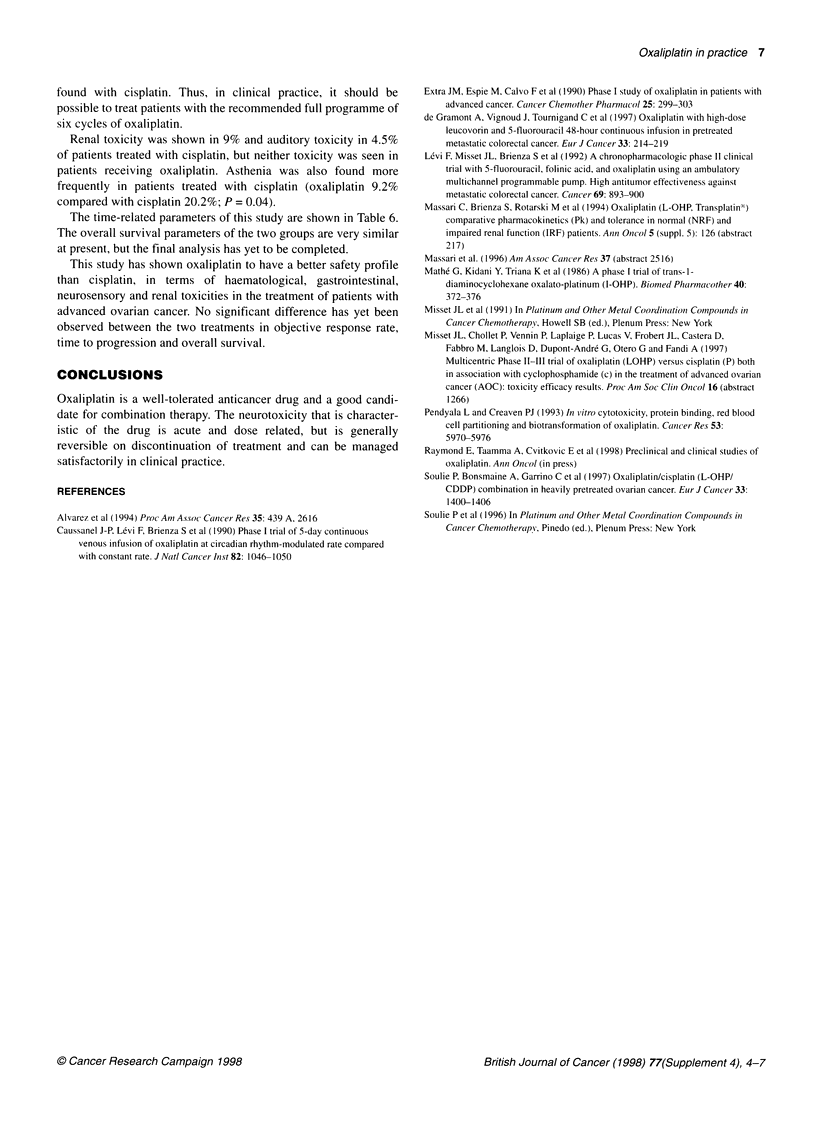# Oxaliplatin in practice.

**DOI:** 10.1038/bjc.1998.428

**Published:** 1998-06

**Authors:** J. L. Misset

**Affiliations:** SMST HÃ´pital Paul Brousse, Villejuif, France.

## Abstract

Oxaliplatin, a new third-generation platinum complex, is active in the treatment of colorectal and advanced ovarian cancers, both as monotherapy and in combination therapy. It has demonstrated a very good safety profile, characterized by low haematotoxicity, and moderate and manageable gastrointestinal toxicity. No significant renal or ototoxicities have been observed. Oxaliplatin induces a peripheral sensory neuropathy which is characterized by distal and perioral dysaesthesia, and is induced or exacerbated by the cold; in general, it is regressive between cycles of treatment. This dose-limiting toxicity is cumulative, but reversible within a few months of discontinuation of treatment in the majority of cases. In a cohort study of 490 patients with advanced colorectal cancer included in an extended access programme, more than 2700 cycles of oxaliplatin plus 5-fluorouracil (5-FU) were administered. The overall safety profile of oxaliplatin was shown to be very favourable. Oxaliplatin and cisplatin, each in combination with cyclophosphamide, have a similar efficacy in the treatment of advanced ovarian cancer, but oxaliplatin was better tolerated than cisplatin in terms of haematological, gastrointestinal, neurosensory and renal toxicities. The safety profile of oxaliplatin makes it an ideal candidate for combination therapy.


British Journal of Cancer (1998) 77(Supplement 4), 4–7
© 1998 Cancer Research Campaign

### Oxaliplatin in practice

**J-L Misset**

SMST Hôpital Paul Brousse, Villejuif, France

**Summary** Oxaliplatin, a new third-generation platinum complex, is active in the treatment of colorectal and advanced ovarian cancers, both as monotherapy and in combination therapy. It has demonstrated a very good safety profile, characterized by low haematotoxicity, and moderate and manageable gastrointestinal toxicity. No significant renal or ototoxicities have been observed. Oxaliplatin induces a peripheral sensory neuropathy which is characterized by distal and perioral dysaesthesia, and is induced or exacerbated by the cold; in general, it is regressive between cycles of treatment. This dose-limiting toxicity is cumulative, but reversible within a few months of discontinuation of treatment in the majority of cases. In a cohort study of 490 patients with advanced colorectal cancer included in an extended access programme, more than 2700 cycles of oxaliplatin plus 5-fluorouracil (5-FU) were administered. The overall safety profile of oxaliplatin was shown to be very favourable. Oxaliplatin and cisplatin, each in combination with cyclophosphamide, have a similar efficacy in the treatment of advanced ovarian cancer, but oxaliplatin was better tolerated than cisplatin in terms of haematological, gastrointestinal, neurosensory and renal toxicities. The safety profile of oxaliplatin makes it an ideal candidate for combination therapy.

**Keywords:** colorectal cancer; ovarian cancer; oxaliplatin; reversible peripheral sensory neuropathy

Oxaliplatin was introduced into clinical trials by Mathé and colleagues in 1986, and has been approved in France for the treatment of advanced colorectal cancer, both as monotherapy and in combination with 5-fluorouracil (5-FU)/folinic acid (FA), for more than a year. To date, more than 2000 patients have received oxaliplatin in clinical trials, 1400 of these for the treatment of metastatic colorectal cancer. All of these studies have confirmed the efficacy and favourable toxicity and safety profiles of oxaliplatin.

Oxaliplatin as monotherapy has also shown activity in pretreated ovarian cancer patients (Misset et al, 1991). The lack of cross-resistance with cisplatin and carboplatin has been demonstrated in human ovarian cancer cell lines, both in vitro and in vivo (Pendyala et al, 1993; Alvarez et al, 1994). This activity profile, together with an excellent tolerability, provides the rationale for the use of oxaliplatin in combination with cisplatin in pretreated ovarian cancer patients. The combinations of oxaliplatin–cisplatin and oxaliplatin–cisplatin–epirubicin–ifosmamide have been shown to be active in such patients (Soulié et al, 1996, 1997).

Oxaliplatin is a non-conventional platinum compound that differs from cisplatin in its lack of nephrotoxicity and from carboplatin in its association with only limited haematological toxicity. The most frequent acute side-effect of oxaliplatin is a transient peripheral neuropathy, manifesting as parasethesia and dysaesthesia in the extremities, which is triggered or enhanced by exposure to cold. These are discussed below.

### TOXICITY PROFILE

### Haematotoxicity

In a large phase I study, 44 patients with advanced cancer received 116 courses of oxaliplatin, with dose escalation from 45 to 200 mg $m^{-2}$ (Extra et al, 1990). Most patients had previously received chemotherapy. Moderate haematological toxicity was observed. Thrombocytopenia was dose related and did not occur at doses of oxaliplatin less than 90 mg $m^{-2}$; however, it did occur in 13% of patients receiving doses of 135–150 mg $m^{-2}$, and 28.5% of those treated with 175–200 mg $m^{-2}$ exhibited a decreased platelet count that did not exceed World Health Organization (WHO) grade 2. Similarly, only grade 1 or 2 neutropenia was observed, and haemoglobulin levels generally remained unchanged.

Table 1 summarizes the incidence of grade 3 and 4 haematotoxicity as a result of the administration of oxaliplatin in combination with 5-FU/FA (Lévi et al, 1992; de Gramont et al, 1997). The 39% grade 3 and 4 neutropenia observed in the Folfox 2 study was manageable, and was a result of 5-FU. It could be avoided in most patients as it almost always occurred after 5-FU dose escalation, and did not recur after further dose adjustment. No haematopoietic growth factors were used.

Thus, oxaliplatin in combination with 5-FU/FA does not increase the haematological toxicity. Indeed, the profile resulting from this treatment combination is almost entirely the result of the 5-FU/FA component of the treatment.

### Renal toxicity

Unlike cisplatin, oxaliplatin does not appear to be nephrotoxic, and its administration does not require any specific nephroprotective measures (e.g. hyperhydration). The pharmacokinetic behaviour of oxaliplatin has been evaluated in patients with normal and impaired kidney function (creatinine clearance value, < 60 ml $min^{-1}$) (Massari et al, 1994; Raymond et al, 1998). Results showed that, after a 2-h infusion of oxaliplatin, no differences were observed between the plasma concentration of the drug in patients with renal impairment and in those with normal renal function, suggesting that dose modification was not required in patients with impaired renal function. Forty-nine patients with impaired renal function have been treated with full-dose oxaliplatin, either as monotherapy or in combination, without evidence of increased nephrotoxicity (Massari et al, 1996).

**Table 1** Comparative haematological toxicity (WHO grades 3 and 4) of oxaliplatin (L-OHP) and oxaliplatin combined with 5-fluorouracil (5-FU) and folinic acid (FA)

| | L-OHP | L-OHP + 5-FU/FA | |
|---|---|---|---|
| | | Folfox 2 | CM |
| Number of patients | 124 | 46 | 93 |
| Anaemia (%) | 3 | 0 | <1 |
| Thrombocytopenia (%) | 2 | 11 | 0 |
| Neutropenia (%) | 1 | 39 | 3 |

CM, chronomodulation.

**Figure 1** The cumulative incidence rate of grade 3 (specific scale) neuropathy in 682 patients receiving oxaliplatin as monotherapy or in combination therapy

### Gastrointestinal toxicity

Nausea and/or vomiting were observed in the majority of patients, and it did not seem to increase with the addition of 5-FU (Table 2). Severe nausea and vomiting were seen in 10% of patients treated with oxaliplatin as monotherapy and in 22% of those treated with oxaliplatin in combination with 5-FU. These symptoms can usually be controlled with supportive measures, including anti-$5HT_3$ medications and loperamide. Diarrhoea appears to be more frequent and severe with longer infusion schedules. Severe diarrhoea occurs in approximately 4% of patients who have received oxaliplatin monotherapy compared with 25.3% of patients who have received oxaliplatin plus 5-FU. The incidence of severe mucositis is dependent on the 5-FU regimen used in the studies.

### Neurotoxicity

Acute neurotoxicity associated with oxaliplatin is common (85–95% of patients) but usually not dose-limiting, occurring within hours of treatment and regressing between treatments. Typical symptoms have included paraesthesias, usually presenting as cold-related dysaesthesias, which were seldom painful or associated with cramps. The majority of these toxicities lasted for 7 days or less, and resolved between treatments. A sporadic, sometimes sudden, and self-limiting laryngopharyngeal dysaesthesia is thought to result from decreased sensitivity of the larynx and pharynx, which causes a feeling of difficulty in breathing or swallowing. Symptoms have resolved spontaneously within hours of onset. Symptoms tended to last longer with successive cycles.

Neurological toxicity scales currently available are generally insufficient for grading the characteristics of the dysaesthesia associated with oxaliplatin use. Consequently, a grading system was developed by Lévi and co-workers that takes into account both intensity and duration of symptoms related to oxaliplatin-induced paraesthesia/dysaesthesia (Caussanel et al, 1990). This is shown in Table 3, in which it is compared with the WHO grading system for neurotoxicity.

**Table 2** Gastrointestinal toxicity found with oxaliplatin as monotherapy and in combination with 5-FU/FA (Massari et al, 1996)

| | Oxaliplatin monotherapy | | | Oxaliplatin plus 5-FU/FA | | |
|---|---|---|---|---|---|---|
| | Number of patients | All grades of toxicity (%) | Grades 3/4 (%) | Number of patients | All grades of toxicity (%) | Grades 3/4 (%) |
| Nausea/vomiting | 262 | 64.9 | 10.7 | 381 | 89.8 | 22.3 |
| Diarrhoea | 250 | 30.4 | 4 | 376 | 84.8 | 25.3 |

**Table 3** The specific grading system of Lévi for neurosensory toxicity compared with the WHO system for grading neurotoxicity (Lévi et al, 1992)

| WHO | Specific scale (after Lévi) |
|---|---|
| 0 Nothing | 0 Nothing |
| 1 Paraesthesias and/or reduction of tendinous reflex | 1 Paraesthesias and/or dysaesthesias (induced by cold) with complete regression within 1 week |
| 2 Severe paraesthesias and/or moderate asthenia | 2 Paraesthesias and/or dysaesthesias with complete regression within 21 days |
| 3 Intolerable paraesthesias and/or reduction of muscular force | 3 Paraesthesias and/or dysaesthesias with incomplete regression at day 21 |
| 4 Paralysis | 4 Paraesthesias and/or dysaesthesias with functional consequence |

© Cancer Research Campaign 1998

**Table 4** Percentage of patients whose neurotoxicity reversed after discontinuation of oxaliplatin treatment

| Neurotoxicity | Time after treatment discontinuation | | | | |
|---|---|---|---|---|---|
| | 1 month | 3 months | 6 months | 9 months | 12 months |
| Total disappearance (%) | 1 | 6 | 18 | 28 | 41 |
| Total and partial disappearance (%) | 6 | 38 | 65 | 76 | 82 |
| No change (%) | 91 | 62 | 35 | 22 | 18 |

**Table 5** Overall toxicity seen in patients treated with oxaliplatin in combination with 5-FU/FA (number of patients, 472; number of cycles, 2645) (data on file)

| | Percentage of patients with grade 3/4 toxicity (% cycles) |
|---|---|
| Haematological toxicity | |
| Anaemia | 8 (2) |
| Leucopenia | 11 (4) |
| Neutropenia | 16 (5) |
| Thrombocytopenia | 5 (1) |
| Gastrointestinal | |
| Nausea/vomiting | 10 (2) |
| Diarrhoea | 17 (5) |
| Mucositis | 6 (1) |
| Neurotoxicity (specific scale) | 11 (6) |
| Other toxicities | |
| Renal | < 1 (< 1) |
| Auditory | – |
| Allergic | < 1 (< 1) |
| Infection | 3 (< 1) |
| Alopecia | 3 (1) |
| Cutaneous | < 1 (< 1) |

A recent safety evaluation in 682 patients, who received either oxaliplatin alone or in combination with 5-FU, indicated that, at a mean cumulative dose of 900 mg $m^{-2}$, 12% of patients experienced grade 3 neurotoxicity, which presented as fine movement disturbances and moderate sensory ataxia (Figure 1) (de Gramont et al, 1997). Further evaluation has shown neuropathy grade 3/4 (Lévi's scale) to occur with oxaliplatin in 75% of patients after 12 cycles and administration of a cumulative dose of 1560 mg $m^{-2}$. However, this cumulative sensory neuropathy is generally reversible after discontinuation of treatment, and disappears entirely at 6–8 months in 42% of patients (Table 4). The incidence of cumulative neurotoxicity was similar for oxaliplatin monotherapy and the combination of oxaliplatin plus 5-FU ± FA. Little or no ototoxicity has been observed.

### SAFETY PROFILE

### Overall safety

In the phase IV Extended Access Programme, patients were treated with oxaliplatin, either as monotherapy or in combination with 5-FU/FA. Safety data from 490 patients and 2702 cycles of treatment have now been evaluated. Some of the patients were heavily pretreated with oxaliplatin, being the fifth line of treatment, with a median cumulative dose of 600 mg $m^{-2}$ (range 70–2300 mg $m^{-2}$). However, the overall safety profile of oxaliplatin in this population of patients proved to be very favourable (Table 5) and confirmed the clinical trial results.

One phase III trial, in which the combination of oxaliplatin plus cyclophosphamide was compared with the combination of cisplatin plus cyclophosphamide, has been completed in patients with advanced ovarian cancer (Misset et al, 1997). The primary objective of this multicentre trial was to evaluate the safety profile of each treatment, and the secondary objectives were to compare efficacy, progression-free survival and overall survival. Patients ($n$ = 182) were randomized to receive cisplatin 100 mg $m^{-2}$ i.v. for 1 h every 3 weeks or oxaliplatin 130 mg $m^{-2}$ i.v. for 2 h every 3 weeks, for six cycles of treatment. Both groups also received cyclophosphamide 1000 mg $m^{-2}$ i.v. for 2 h every 3 weeks.

An interim analysis of the safety data was performed and the results showed that the treatment in both arms of the study was acceptable, with over 450 cycles of treatment being administered in each group. It was found that almost 90% of the planned dose of oxaliplatin could be given. The cisplatin dose was reduced to 85.7% because of haematotoxicity. In addition, the dose of cyclophosphamide was reduced further in this group, to 85% compared with 90.5% in the group receiving oxaliplatin.

Haematotoxicity of grade 3/4 caused a delay in twice the number of cisplatin treatment cycles compared with oxaliplatin (141 compared with 70 for all grades of haematotoxicity: $P$ = 0.001). Gastrointestinal toxicity was also lower in the group of patients receiving oxaliplatin, with high-grade nausea and vomiting occurring in a significantly smaller proportion of patients (oxaliplatin 7.9% compared with cisplatin 25.9%: $P$ = 0.001).

When the levels of neurosensory toxicity were investigated, it was found that during the early stages of the trial, low-grade toxicity occurred much more frequently in the group of patients treated with oxaliplatin. By the end of the therapeutic programme, however, there was a complete absence of the anticipated grade 3 toxicity in this patient group, while cumulative neurotoxicity was

**Table 6** Time-related parameters

| | Cisplatin (+ cyclophosphamide) | Oxaliplatin (+ cyclophosphamide) | Significance[a] |
|---|---|---|---|
| Median survival (days) | 770 | 782 | $P$ = 0.83 |
| (range) | (12–1680+) | (39–1567+) | (NS) |
| Median time to progression (days) | 432 | 412 | $P$ = 0.6 |
| (range) | (12–1426+) | (6–1524+) | (NS) |

[a]$\chi^2$.

© Cancer Research Campaign 1998

found with cisplatin. Thus, in clinical practice, it should be possible to treat patients with the recommended full programme of six cycles of oxaliplatin.

Renal toxicity was shown in 9% and auditory toxicity in 4.5% of patients treated with cisplatin, but neither toxicity was seen in patients receiving oxaliplatin. Asthenia was also found more frequently in patients treated with cisplatin (oxaliplatin 9.2% compared with cisplatin 20.2%; $P = 0.04$).

The time-related parameters of this study are shown in Table 6. The overall survival parameters of the two groups are very similar at present, but the final analysis has yet to be completed.

This study has shown oxaliplatin to have a better safety profile than cisplatin, in terms of haematological, gastrointestinal, neurosensory and renal toxicities in the treatment of patients with advanced ovarian cancer. No significant difference has yet been observed between the two treatments in objective response rate, time to progression and overall survival.

### CONCLUSIONS

Oxaliplatin is a well-tolerated anticancer drug and a good candidate for combination therapy. The neurotoxicity that is characteristic of the drug is acute and dose related, but is generally reversible on discontinuation of treatment and can be managed satisfactorily in clinical practice.

### REFERENCES

Alvarez et al (1994) *Proc Am Assoc Cancer Res* **35**: 439 A, 2616

Caussanel J-P, Lévi F, Brienza S et al (1990) Phase I trial of 5-day continuous venous infusion of oxaliplatin at circadian rhythm-modulated rate compared with constant rate. *J Natl Cancer Inst* **82**: 1046–1050

Extra JM, Espie M, Calvo F et al (1990) Phase I study of oxaliplatin in patients with advanced cancer. *Cancer Chemother Pharmacol* **25**: 299–303

de Gramont A, Vignoud J, Tournigand C et al (1997) Oxaliplatin with high-dose leucovorin and 5-fluorouracil 48-hour continuous infusion in pretreated metastatic colorectal cancer. *Eur J Cancer* **33**: 214–219

Lévi F, Misset JL, Brienza S et al (1992) A chronopharmacologic phase II clinical trial with 5-fluorouracil, folinic acid, and oxaliplatin using an ambulatory multichannel programmable pump. High antitumor effectiveness against metastatic colorectal cancer. *Cancer* **69**: 893–900

Massari C, Brienza S, Rotarski M et al (1994) Oxaliplatin (L-OHP, Transplatin®) comparative pharmacokinetics (Pk) and tolerance in normal (NRF) and impaired renal function (IRF) patients. *Ann Oncol* **5** (suppl. 5): 126 (abstract 217)

Massari et al. (1996) *Am Assoc Cancer Res* **37** (abstract 2516)

Mathé G, Kidani Y, Triana K et al (1986) A phase I trial of trans-1-diaminocyclohexane oxalato-platinum (I-OHP). *Biomed Pharmacother* **40**: 372–376

Misset JL et al (1991) In *Platinum and Other Metal Coordination Compounds in Cancer Chemotherapy*, Howell SB (ed.), Plenum Press: New York

Misset JL, Chollet P, Vennin P, Laplaige P, Lucas V, Frobert JL, Castera D, Fabbro M, Langlois D, Dupont-André G, Otero G and Fandi A (1997) Multicentric Phase II–III trial of oxaliplatin (LOHP) versus cisplatin (P) both in association with cyclophosphamide (c) in the treatment of advanced ovarian cancer (AOC): toxicity efficacy results. *Proc Am Soc Clin Oncol* **16** (abstract 1266)

Pendyala L and Creaven PJ (1993) *In vitro* cytotoxicity, protein binding, red blood cell partitioning and biotransformation of oxaliplatin. *Cancer Res* **53**: 5970–5976

Raymond E, Taamma A, Cvitkovic E et al (1998) Preclinical and clinical studies of oxaliplatin. *Ann Oncol* (in press)

Soulie P, Bonsmaine A, Garrino C et al (1997) Oxaliplatin/cisplatin (L-OHP/CDDP) combination in heavily pretreated ovarian cancer. *Eur J Cancer* **33**: 1400–1406

Soulie P et al (1996) In *Platinum and Other Metal Coordination Compounds in Cancer Chemotherapy*, Pinedo (ed.), Plenum Press: New York

© Cancer Research Campaign 1998